# Supplementary material for: Targeting Lysosomes to Reverse Hydroquinone-Induced Autophagy Defects and Oxidative Damage in Human Retinal Pigment Epithelial Cells
Source: Int J Mol Sci. 2021 Aug 22;22(16):9042. doi: 10.3390/ijms22169042 (PMC8396439; doi:10.3390/ijms22169042)
Supplement: Supplementary file 1 [file ijms-22-09042-s001.zip › ijms-1323391-supplementary.pdf]

Supplementary data

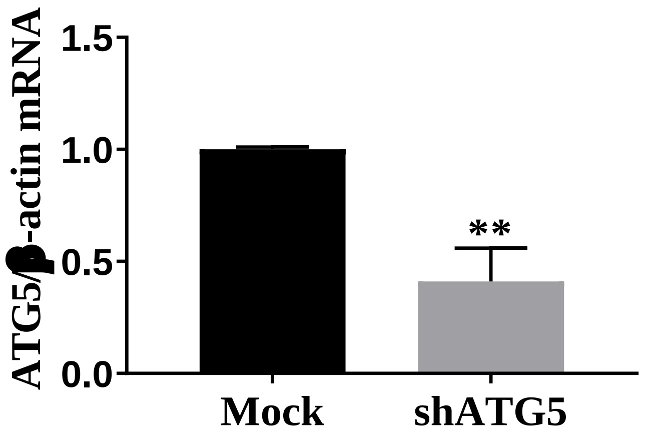

A

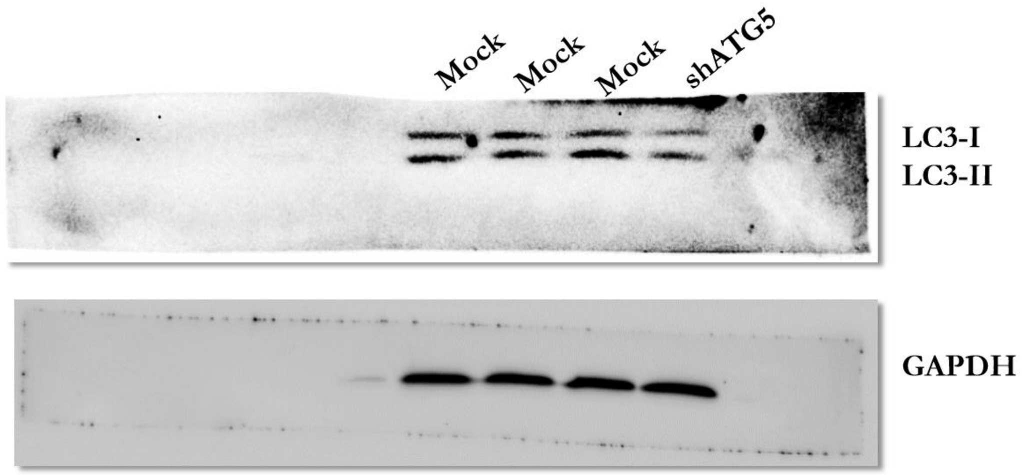

B

Figure S1. Transfection of cells with lentivirus particles coding shRNA ATG5 or scrambled RNA.

(A) Quantitative RT-PCR to quantify ATG5 levels in ARPE-19 cells after transfection.

(B) Immunoblot for LC3-II in whole-cell lysates after transfection.
